# Antibodies and Brain Disease: A Convergence of Immunology and Physiology

**DOI:** 10.1371/journal.pmed.0030498

**Published:** 2006-12-19

**Authors:** Betty Diamond, Bruce T Volpe

## Abstract

The authors discuss a new study, by Emmer and colleagues, which attempted to test whether the presence of an antibody called anti-NMDAR correlates with brain abnormality in patients with SLE.

Increasingly, patients with systemic lupus erythematosus (SLE) are recognized to have central nervous system (CNS) manifestations. Overt inflammation in the CNS is rare, but impairment of cognition or mood disturbance is common. Treatment of inflammation is directed at the underlying autoimmune disorder; treatment of other manifestations, including mood disorder, often focuses on symptom alleviation. Cognitive impairment in particular appears to be progressive and unrelated to disease activity, and, at the present time, untreatable. One interesting aspect of neuropsychiatric lupus is that symptoms may remit as well as progress.

With no known pathophysiology for the cognitive impairment or mood disturbance seen in SLE, and no ability to track the dynamic state of either of these symptom complexes, it has been difficult to develop a meaningful classification of neuropsychiatric SLE or a mechanistic understanding of these aspects of the disease process. There is possible mechanistic precedent for neuropsychiatric lupus in a number of disease syndromes in which anti-neuronal antibodies cause damage in the CNS. Often these antibodies arise as a consequence of microbial infection and cross-react with microbial antigens, as in post-streptococcal movement disorders [1], post-infectious polyradiculopathies [2], and tropical spastic paraparesis [3].

## Anti-Neuronal Antibodies in SLE

Recently, a novel pathophysiology for cognitive decline and mood disorder in SLE has been proposed: cross-reacting anti-DNA, anti-N-methyl-D-aspartate receptor (NMDAR; see Glossary) antibodies mediating neuronal damage or death. These antibodies bind a 5–amino acid epitope D/E W D/E Y S/G in the extracellular domains of the NR2A and NR2B subunits of the NMDAR [4–9].

**Glossary**

**D/E W D/E Y S/G:** A 5–amino acid consensus sequence recognized by lupus antibodies and present in the NR2A and NR2B subunits of the NMDA receptor.

**DWEYS peptide:** The antigen used in enzyme-linked immunosorbent assays to look for anti-NMDA receptor antibodies.

**Epitope:** An antigenic determinant recognized by an antibody molecule.

**N-methyl-D-aspartate receptor:** A receptor for a neurotransmitter critically involved in learning and memory, and present in high density in the hippocampus and amygdala.

Emerging clinical information about the frequency of these anti-DNA, anti-NMDAR antibodies in patients with SLE suggests that 30%–50% of patients have these antibodies [10–14]. In one longitudinal study, brain dysfunction correlated with the presence of antibody to DWEYS peptide in the cerebrospinal fluid, and symptom severity correlated with antibody titer [10]. But cross-sectional analyses of neuropsychological function and serum anti-NMDAR antibody levels have yielded conflicting data [11–13]. Two studies found a significant correlation between anti-NMDAR antibodies and depression [12,13] and one study found a correlation with neuropsychiatric disease manifestations [10]. Another study failed to show a correlation between anti-NMDAR antibodies and disease manifestations [11]; however, these investigators required more extensive cognitive impairment for diagnosis.

## A New Technique to Study Neuronal Damage

In a new study in *PLoS Medicine*, Emmer and colleagues attempted to test whether the presence of the anti-NMDAR antibody correlates with brain abnormality [15]. The abnormality was assessed by an emerging quantitative magnetic resonance imaging technique—diffusion weighted images (DWIs) and the use of the apparent diffusion coefficient (ADC). DWIs and the ADC offer the promise of a quantitative physiological measure of cellular integrity from magnetic resonance images. ADCs measure the movement of water molecules that are systematically altered in circumstances of subacute, acute, and chronic tissue inflammation, injury, and destruction. Using various magnetic resonance stimulation data acquisition and processing schemes, an ADC is generated—“apparent” because diffusion in vivo is inhibited by cellular structures, membranes, and other molecules.

Pioneering studies of patients with multiple sclerosis (MS) and stroke, for example, have recently shown quantitative structure–function relationships between the ADC and cognitive impairment in MS [16], and the ADC and various states of stressed ischemic brain tissue in stroke [17]. Based on information gathered from studies of stroke, it would appear that the regional vascular compromise of stroke produces a breakdown in energy generation, an increase in intracellular (cytotoxic) edema, and a shrinkage of the extracellular compartment, leading to decreased ADC and hyperintense DWIs. This abnormal physiology may resolve coincident with the return over days of ADC values to normal. In chronic MS, there is a loss of tissue integrity with increased vasogenic edema and high ADC. In contrast, an acute intense inflammatory reaction with increased microglia simultaneously stressing and destroying neurons causes a fall in diffusion and the ADC.

## Using the ADC to Study Neuropsychiatric Lupus

Currently, there are two published studies that used the ADC to study patients with SLE. One is by some of the authors of the new study in PLoS Medicine. Their results showed that compared to controls, all 11 patients with SLE and CNS involvement had higher mean ADC values, suggesting loss of tissue integrity, perhaps caused by the chronic degenerative effects of stroke (*n* = 4) or seizures (*n* = 3) and atrophy [18]. Four of 20 patients in the other study had decreased ADC that correlated with focal ischemic regions; four had increased ADC and chronic small strokes from severe hypertension [19].

In contrast, Emmer at al. now show that compared to 12 healthy control patients and 21 patients with SLE and no symptoms referable to the CNS, 37 patients with neuropsychiatric lupus had decreased mean ADC in the hippocampus and amygdala [15]. Of the 37 patients with neuropsychiatric lupus, 11 had strokes, nine had cognitive dysfunction, two had acute confusional states, six had mood or anxiety disorders, one had psychosis, and the others had neuropathy, myelopathy, or headache syndromes.

Additionally, the authors show that ADC measured across all forebrain grey and white matter is comparable in patients with or without disease, yet there was regional depression of the ADC in the hippocampus and amygdala. In a small subgroup of patients with anti-NMDAR antibodies (*n* = 4), the ADC was further depressed in the amygdala compared to patients without anti-NMDAR antibodies. It remains to be determined whether decreased ADC in the context of magnetic resonance structural images that show a normal hippocampus and amygdala represents a marker of neuron stress, as in ischemia, or acute inflammation as in MS, or both. Nevertheless, the application of these advanced imaging techniques to specific brain regions is remarkable in comparison to these authors' earlier findings [18]. The regionally specific depressed ADC confined to the hippocampus and amygdala is obviously interesting in view of the high concentration of NMDAR in these regions, and the preliminary studies that show a correlation of depression and cognitive impairment with anti-NMDAR antibodies.

## Conclusion

At this point the data are tantalizing but scant. They do not permit strong prediction about the verity of the murine model, which depends not only on the neurotoxicity of cross-reacting anti-DNA, anti-NMDAR antibodies, but also on the ability of the anti-NMDAR antibodies to gain access to brain tissue. Because the magnetic resonance techniques used in this study are low risk and have the capacity to capture phases of a dynamic physiological process, the techniques promise to assist substantially in deciphering the pathogenicity of neuropsychiatric lupus.

## Abbreviations

ADC: apparent diffusion coefficient
CNS: central nervous system
DWI: diffusion weighted image
MS: multiple sclerosis
NMDAR: N-methyl-D-aspartate receptor
SLE: systemic lupus erythematosus
